# Influence of Selective Laser Melting Machine Source on the Dynamic Properties of AlSi10Mg Alloy

**DOI:** 10.3390/ma12071143

**Published:** 2019-04-08

**Authors:** Ben Amir, Shmuel Samuha, Oren Sadot

**Affiliations:** 1 Department of Mechanical Engineering, Ben-Gurion University of the Negev, Beer-Sheva 84105, Israel; ben333amir@gmail.com; 2 Department of Materials Engineering, NRCN, P.O. Box 9001, Beer-Sheva 84190, Israel; samuha@post.bgu.ac.il

**Keywords:** selective laser melting, AlSi10Mg alloy, dynamic properties, impact, crystallographic texture

## Abstract

Selective laser melting (SLM) AlSi10Mg alloy has been thoroughly investigated in terms of its microstructure and quasi-static properties, owing to its broad industrial applications. However, the effects of the SLM process on the dynamic behavior under impact conditions remain to be established. This research deals with the influences of manufacturing process parameters on the dynamic response of the SLM on AlSi10Mg at a high strain rate of 700 to 6700 s^−1^ by using a split Hopkinson pressure bar apparatus. Examinations were performed on vertically and horizontally built samples, processed individually by two manufacturers using a different laser scanning technique on the same powder composition. It was concluded that the fabrication technique does not influence the true stress–true strain dependency at strain rates of 700 to 2800 s^−1^. However, at higher strain rates (4000 to 6700 s^−1^), this study revealed different plastic behavior, which was associated only with the horizontally built samples. Moreover, this study found different failure demeanors at true strains exceeding 0.8. The dynamic response was correlated with the as-built microstructure and crystallographic texture, characterized using the electron backscattered diffraction technique.

## 1. Introduction

Additive manufacturing (AM) is an advanced fabrication technique in which a component is built up in successive layers of material to create a three-dimensional (3D) structure under a computer control system. The process can be conducted from a wire feedstock, metallic sheets, or a powder that is selectively melted or sintered using an energy source [1]. In the selective laser melting (SLM) process, a thin powder bed layer is scattered on top of the previous melted layer. A laser beam selectively melts the powder at the locations that need to be solidified. The thickness of each powder layer is approximately 20 to 60 µm [2]. This step is followed by the depositing of fresh powder. The laser melts the new powder and a partial volume of the previous layer to consolidate the two layers together. This process continues until the part is completed [3].

Aluminum alloys are of significant interest for lightweight applications in the aerospace and automotive sectors. In particular, AlSi10Mg alloy is compatible with SLM as it is close to a eutectic Al–Si composition, promoting effective castability and strong weldability, as well as exhibiting very high density (99.7% pure aluminum). Moreover, age hardening is enabled by Mg and Fe, which increases the strength by means of the Mg_2_Si and Al5FeSi precipitation sequence [3,4,5,6,7]. For SLM–ALSi10Mg, the enhanced or at least similar mechanical properties compared to conventional high-pressure die-cast AlSi10Mg material can be attributed to the extremely fine microstructure resulting from the manufacturing process [3,8,9,10,11,12]. Numerous process parameters in SLM directly or indirectly alter the final product, and therefore, various studies have attempted to determine the parameters with the greatest impact on the manufacturing process. Some of the parameters that have been identified are the laser power, scanning velocity, hatching distance, and scanning pattern. Substantial efforts have been made to optimize the processing parameters to obtain similar or even superior mechanical properties with respect to cast AlSi10Mg alloy [9,13,14,15]. It has been suggested that the hatching distance has a significant effect on the mechanical properties and porosity, by increasing the volumetric energy, the porosity can be reduced at the scanning center and increased at scan edges [12,13,16]. The volumetric energy porosity together with the scanning strategy affects the surface topology, dimensional accuracy, residual stresses, and crystallographic texture [14,15,17]. In order to optimize the fatigue resistance, reduction of the porosity must occur mainly at the surface or the subsurface of the samples, along with post-processing heat treatment [3,16]. In addition to optimization research, extensive inquiries into the quasi-static mechanical properties of SLM aluminum alloy have been conducted [3,4,6,18,19,20,21]. These studies have suggested that the SLM aluminum yield stress, elongation, and hardness are approximately the same or even higher than those of conventional cast aluminum.

A study performed by Rosenthal et al. revealed sensitivity to the strain rate in the range of 2.77 × 10^−6^ to 2.77 × 10^−1^ s^−1^ [6]. Moreover, they and others identified a noticeable dependency of the mechanical properties on the building orientation under quasi-static stress–strain conditions [17,18].

To date, the majority of studies have focused on the quasi-static mechanical properties of SLM–AlSi10Mg alloy. However, to the best of the authors’ knowledge, a serious gap exists in the available literature regarding the mechanical behavior of SLM–AlSi10Mg alloy under high strain rates. It should be emphasized that it is imperative to investigate the dynamic behavior of SLM–AlSi10Mg alloy, as unpredicted impact situations in service conditions (such as accidental events including vehicle crashes) may expose this alloy to dynamic loading.

Recent studies by Asgari et al. [22] and Hadadzadeh et al. [23] investigated the behavior of SLM–AlSi10Mg alloy under dynamic loading in a strain rates range of approximately 150 to 4300 s^−1^, and noted that increasing the strain rate results in a higher dynamic yield, as well as modification of the alloy substructure and crystallographic texture. Moreover, the compressive deformation at a strain rate of 1400 s^−1^ exhibits an increase in the dislocation density and development of accumulated dislocation networks. However, no dependency in the building orientation was identified [24].

In an early study by our group [8], we focused on the effect of heat treatment on the dynamic mechanical properties. In [8], the investigated SLM–AlSi10Mg alloy was fabricated by EOS (described in Section 2) using a similar set of SLM parameters as used in the present study, under strain rates ranging from 700 to 7900 s^−1^. It was found that the dynamic behavior is sensitive to heat treatment and varies drastically as a function of the building orientation. Nurel et al. [8] noted that the heat treatment reduced the dynamic behavior differences but did not eliminate it. Moreover, it was shown that the strain rate had a minor effect on the mechanical properties. Moreover, Zaretsky et al. characterized the dynamic properties of SLM–AlSi10Mg, under strain rates ranging from 5000 to 100,000 s^−1^, using planar impact tests and found that the dynamic response of SLM-processed alloys is virtually independent of the processing orientation [25].

To the best of the authors’ knowledge, the dynamic response of SLM–AlSi10Mg alloy has been studied on samples that differed only in their built-up direction, regardless of the pattern strategy and other process parameters. Thus, the novelty of the present work is to provide an improved understanding of the roles of the process parameters and building direction on the dynamic properties of AlSi10Mg alloy. For this purpose, the samples were fabricated by the EOS M280 and the Concept Laser (CL) X line 1000R. Both of these implement similar SLM-powder bed technology using AlSi10Mg alloy and known for their unique fabrication strategy. The dynamic properties were experimentally characterized using the split Hopkinson pressure bar (SHPB) on samples in the “as-built” state (without heat treatment), at two different building orientations, XY- and Z-oriented, under strain rates ranging from 700 to 6700 s^−1^—which were achieved by impact velocities in the range of 10–30 m/s, similar to those encountered in car accidents. It should be clarified upfront that the aim of this research is not to determine the “best printer”, but rather to understand the manner in which the printing parameters affect the dynamic mechanical properties. Therefore, we chose to test samples manufactured by each machine in its optimal setup. This study combines a systematic investigation of the microstructure and crystallographic texture in the initial state, employing the electron back-scattered diffraction (EBSD) technique coupled with a high-resolution scanning electron microscope (SEM). The remainder of this paper is arranged as follows—the experimental and building methods are described, followed by characterization of the sample microstructures and the results from the SHPB systems. Finally, concluding remarks are provided.

## 2. Experimental Method

### 2.1. Machines and Material

Two machines, distinguished by their unique processing strategies, were selected—the EOS M280 and CL X line 1000R. Each machine fabricated samples of AlSi10Mg alloy using fresh powder, with the chemical compositions presented in Table 1. A comparison between the manufacturer powder particle size and sphericity is presented in Table 2 as a cumulative distribution of the undersized 10%, 50%, and 90% of the tested powder as reported by the manufacturers. In the SLM process, the particle size and the sphericity reflect on the powder quality and have a major influence on the product’s mechanical properties [26]. The slight variation in the fresh powder was considered negligible in the present study.

**Figure 1:**
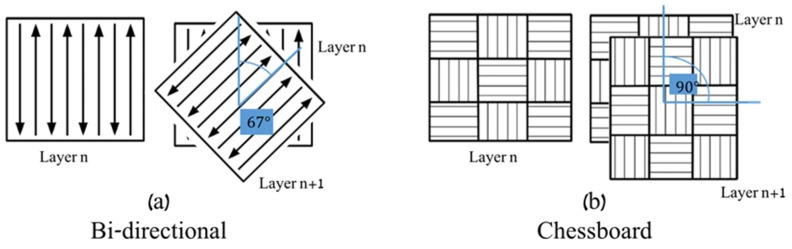
Schematic of scanning strategies by (**a**) bi-directional, EOS M280; and (**b**) chessboard, CL X line 1000R.

The EOS M280 and the CL X line 1000R machines are both fiber laser based SLM systems, distinguished by the set of process parameters, the main parameters are presented in Table 3. Other process parameters can be found on the manufacturer’s web pages [27,28]. The exact set of parameters used to fabricate the samples for the present study was the optimal default setting recommended by the manufacturers. The scanning strategy is different between the two machines. EOS uses the bi-directional scanning strategy of rotating each layer by 67°, as illustrated in Figure 1a; while the “chessboard strategy” built-up process of the CL machine included a 90° rotation between each layer, as illustrated in Figure 1b. The quasi-static tensile mechanical properties of the alloy are presented in Table 4.

### 2.2. Description of Samples

**Figure 2:**
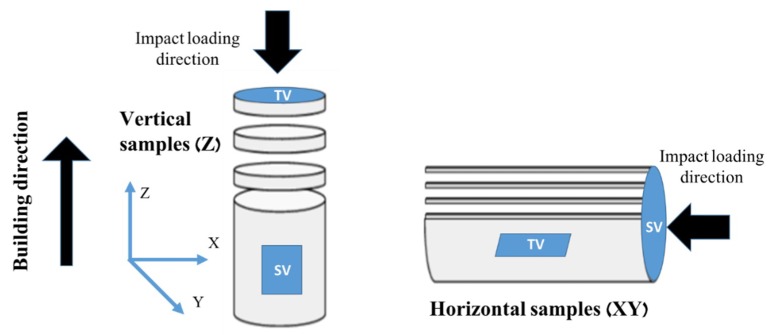
Schematic of vertical and horizontal samples, describing building direction, impact loading direction, and sectioning for microstructural studies. Top view (TV) and side view (SV) denote top view and side view, respectively.

The samples used for the high strain rate tests were machined in two orientations. The vertically built samples, designated by ‘Z’, had their long axes oriented parallel to the building direction; while the horizontally built samples, designated by ‘XY’, were oriented normal to the building direction. The vertical (Z) and horizontal (XY) rod-shaped samples are illustrated in Figure 2. To cancel shell effects and make the standard sample size suitable for our SHPB system, the samples were machined from circular rod-shaped cylinders of 13 mm in diameter and 100 mm in height into circular discs of 7 mm in diameter and 3.5 or 7 mm in height.

### 2.3. SHPB System

**Figure 3:**
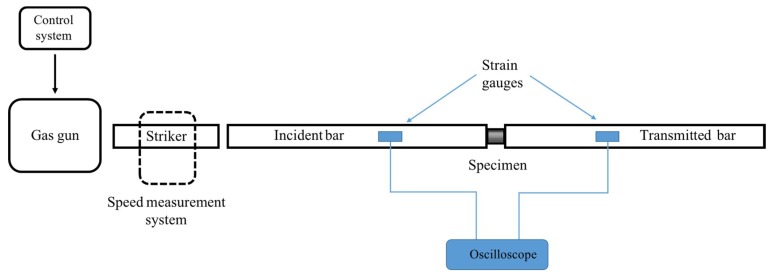
Schematic of the split Hopkinson pressure bar (SHPB) experimental system.

**Figure 4:**
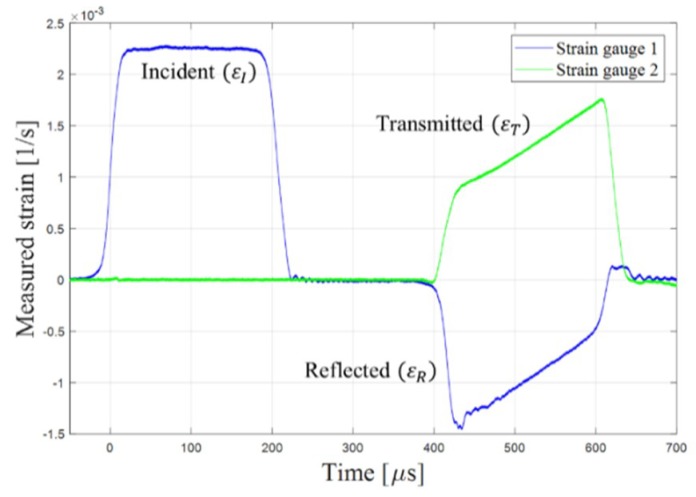
Typical strain signals measured by strain gauges on incident bars (blue) and transmitted bars (green).

Each sample was dynamically loaded using the SHPB system, located at the Shock Wave Laboratory at the Ben Gurion University of the Negev. Figure 3 presents a schematic of the system. The experiment was conducted by placing a sample between the incident and transmission bars. A third, shorter striker bar was accelerated using a gas gun. The striker bar impacted the incident bar, which generated a stress pulse in the incident bar. The striker bar velocity dictated the pressure pulse in the bars. A micro-controller filled the gas gun pressure chamber according to the desired striker velocity. The striker bar velocity was measured in close proximity to the incident bar. The impact between the two bars generated an elastic stress wave that propagated along the incident bar towards the sample. Owing to the acoustic impedance mismatch between the bar and sample, some of the incident pressure waves were reflected back into the incident bar, while the remainder were transmitted into the transmitted bar. The stress wave caused a strain along the bars, which was measured by strain gauges located on the incident and transmission bars. Figure 4 depicts the typical strain signals measured on the two bars. The curves representing the strain signal measured on the incident bar and on the transmitted bar are marked by blue and green lines, respectively. The sample stress, strain, and strain rate were analytically calculated by using the standard SHPB, as expressed in Equations (1)–(3) [29], under the assumptions of a one-dimensional (1D) single-wave analysis and force equilibrium at the specimen surfaces.

(1) $${\dot{\varepsilon }}_{s}(t)=\frac{-2C_{B}\varepsilon_{R}\left(t\right)}{H_{s}}$$

(2) $$\varepsilon_{s}\left(t\right)=-2\frac{C_{B}}{H_{s}}\int_{0}^{t}\varepsilon_{R}\left(t\right)\;dt$$

(3) $$\sigma_{s}(t)=\frac{A_{B}}{A_{s}}E_{B}\varepsilon_{T}(t)$$

The subscripts *S* and *B* refer to the specimen and bar, respectively, while the subscripts *R* and *T* denote the reflected and transmitted bars, respectively. Moreover, *ε* is the strain, $\dot{\varepsilon }$ is the strain rate, *σ* is the stress, and *A* is the area cross-section. *H* is the specimen height, *C_B_* is the elastic wave velocity in the bar, and *E_B_* is the material elastic modulus of the bars.

Hence, in every experiment, the equilibrium assumption was validated by testing that the strain signal created by the incident stress wave (*ε_T_*) was equal to the combined signals created by the transmitted wave (*ε_I_*) and reflected wave (*ε_R_*).

**Figure 5:**
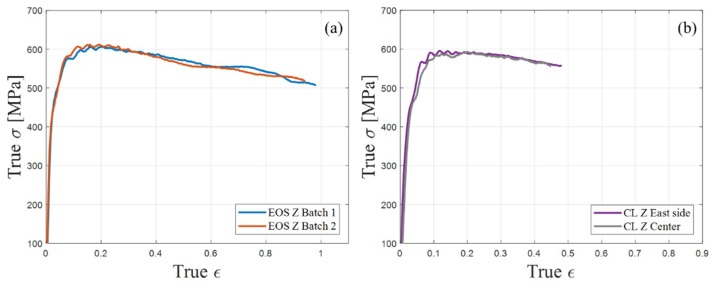
Presentation of the repeatability by comparing, (**a**) 2 EOS‘s Z-orientation samples from deferent batches and, (**b**) CL’s Z orientation samples located at the center and in the east side of the building platform.

The strain rates ranged from 700 to 6700 s^−1^. The experiments were conducted under two strain rates ranges of 700 to 2800 s^−1^ and 4000 to 6700 s^−1^ for each manufacturer (EOS and CL) for each orientation (XY and Z). Each unique experiment was repeated at least three times to ensure reliability and repeatability, 27 experiments were conducted in total. In the present study, the results were presented as the true stress, true strain, and true strain rate, and conversions were performed as detailed in [30]. To confirm that the mechanical properties extracted from the SHPB experiments were reflected solely in the properties of a sample, regardless of its geometry, two sample types distinguished by their geometry were investigated under the same strain rate conditions. The sample heights were 3.5 and 7 mm, with a similar diameter. The relative difference between the two results was less than 1%, and therefore negligible [8]. In order to assure the repetition of the SLM process and its effect on the dynamic properties, two individual batches of samples were fabricated by EOS at Z orientation and tested at the SHPB system. The experiments were performed at strain rates of 4000 to 4300 s^−1^. The true stress–true strain curves are presented in Figure 5a. The difference between the curves is less than 3% and could be considered to be minor. Figure 5b presents Z-oriented samples fabricated by CL in center and on the east side of the building platform. The difference between the results is less than 4%. Therefore, the effect of the sample’s location on the building platform was not taken into consideration. It is well known that 2D/3D effects may exist in SHPB systems that need to be accounted for in certain cases [29]. Such effects cause a dispersion phenomenon, which was investigated and found to be negligible in our system. For further details, see [8].

### 2.4. Microstructural Investigation

The microstructures and crystallographic textures of the as-built samples were studied using SEM (MIRA-3, Tescan) with a field-emission gun, equipped with an EBSD detector. The sample surface preparation included mechanical grounding with SiC papers of grit sizes down to 4000, followed by mechanical-chemical polishing up to 0.05 µm using colloidal silica. The principal directions of the SEM-EBSD samples are denoted by the building direction (BD), transverse direction (TD), and normal direction (ND). Successful interpretation of the Kikuchi patterns was obtained by scanning using a step size ranging from 0.5 to 1 µm, in accordance with the severity of the local deformations and grain sizes. The MTEX package, which is a freely available MATLAB toolbox (2017b), was employed for post-processing and visualization of the EBSD data [31]. The characterization also included studying the specimen fracture surfaces following dynamic testing using a standard stereoscope.

## 3. Results and Discussion

### 3.1. Microstructural Investigation of As-Built Samples

**Figure 6:**
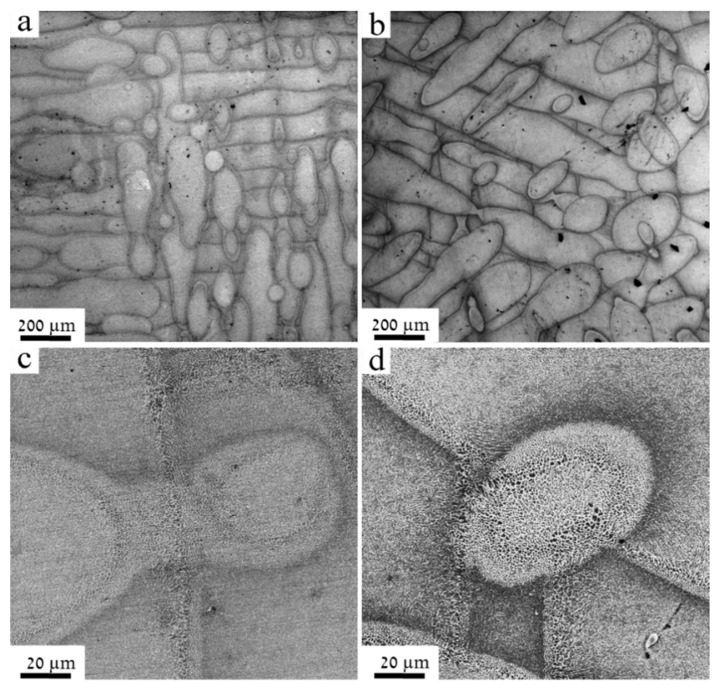
Building surface of SLM–AlSi10Mg part manufactured along the Z orientation, using (**a**) CL and (**b**) EOS techniques, respectively. Images (**c**) and (**d**) are enlarged micrographs of (**a**) and (**b**), respectively.

Figure 6a,b presents top-view images of the fabricated SLM–AlSi10Mg parts, when employing the CL and EOS manufacturing techniques, respectively. For the sake of clarity, enlarged images are presented in Figure 6c,d for the CL and EOS parts, respectively. The unique scan pattern attributed to each manufacturer can be clearly observed. The building surface of the CL part was constructed using an island strategy with 90° rotation (chessboard orientation), as indicated in Figure 6a. A laser-scanning pattern with 67° rotation can be observed in the EOS part in Figure 6b. Typical characteristics of scan vectors and melt pool morphology are evident. It is worthwhile noting the existence of the relatively large pores (in the order of 5 to 50 µm), for the EOS sample. The formation of these pores might be attributed to the process parameters, namely, the rates of scan speed and the hatch spacing, in particular [17,32]. Following a close examination, unmelted powder at the pores’ circumference was not observed, suggesting that these microstructure defects can be designated as metallurgical pores rather than keyhole type. These metallurgical pores nucleate at slow scanning speeds from gases trapped within the melt pool or evolved from the powder during consolidation.

**Figure 7:**
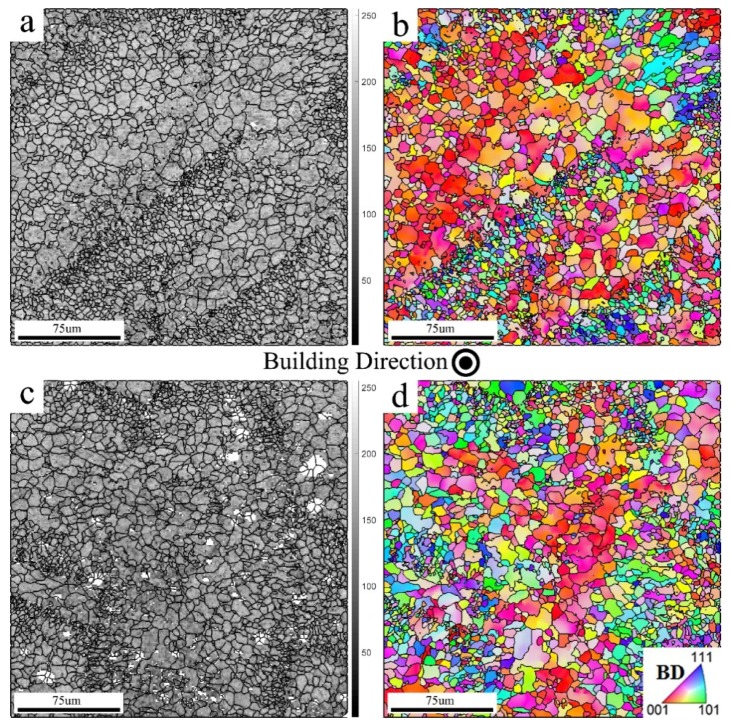
Building surfaces of (**a,b**) CL and (**c,d**) EOS manufactured AlSi10Mg SLM vertical samples, indicating (**a**,**c**) band contrast (BC) and (**b**,**d**) inverse pole figure (IPF) maps, respectively. In (**d**), an inset color-coded key triangle indicates the crystallographic orientations.

Figure 7a,c presents top views of the SLM–AlSi10Mg alloy manufactured by the CL and EOS techniques, respectively. The figures are described using band contrast (BC) maps. In this type of map, the contrast is based on the quality of an EBSD pattern obtained from each pixel. As an example, low BC values (blackish color) are assigned to grain boundary or highly deformed regions, while high BC values (bright color) are assigned to dislocation-free zones. Following a close examination of these maps, it could be observed that both structures consisted of equiaxed grains with bimodal grain sizes. Fine grains were formed at the melt pool boundaries, that is, at the borders of scan edges. Their sizes are attributed to rapid solidification, which in turn hinders the grain growth mechanisms. However, a coarser grain size was present at the melt pool, owing to the localized heat flow. Figure 7b,d presents color-coded inverse pole figure (IPF) maps. These types of maps describe the crystal orientation of a grain relative to the sample coordination system. Similar colors indicate grains with close crystallographic orientations. High-angle grain boundaries (misorientation angles higher than 15°) are highlighted in IPFs using black color marks. Upon investigation of these maps, it was found that coarse grains, present at the melt pool centerline, are oriented preferably with their <001> parallel to the BD (cube texture). This dominant texture component—designated using reddish grains in the IPFs—was expected, as the preferred growth direction of cubic crystals during solidification is <100> [14]. However, the fine grains, present at the melt pool boundaries, deviated far from this texture component, as evidenced by the large variety of colors assigned to these grains.

It is interesting to note that a weaker cube texture was observed for the EOS sample compared to that of the CL. It is plausible to assume that the scanning strategy was the cause of these textural changes. According to [18], a significantly weaker textural component is expected for an SLM product fabricated using a scanning pattern with an axial rotation other than 90°, as in the case of the EOS with a 67° rotation.

**Figure 8:**
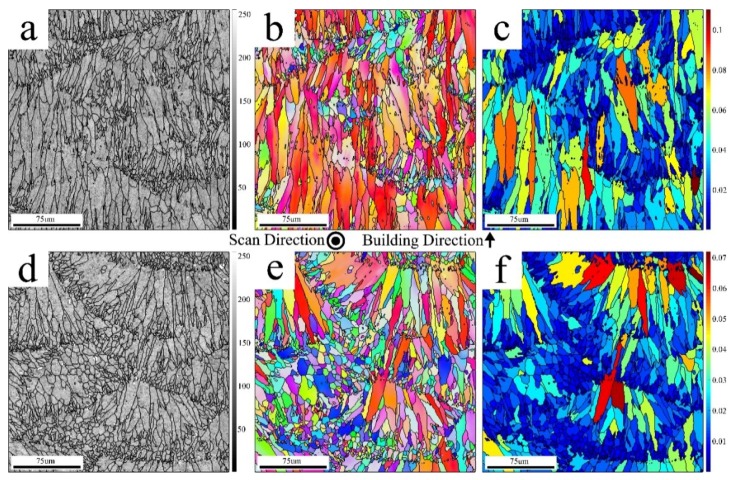
Side view of the SLM–AlSi10Mg samples manufactured by (**a–c**) CL and (**d–f**) EOS, indicating (**a**,**d**) BC, (**b**,**e**) IPF (||BD), and (**c**,**f**) grain orientation spread (GOS) maps, respectively. GOS values are located on the right side of each GOS map.

A side view of the manufactured SLM–AlSi10Mg alloy is presented in Figure 8a–c and Figure 8d–f for CL and EOS, respectively. This plane describes the microstructure and crystallographic texture perpendicular to the building layers. As such, the cross-section of the melt pools is visible, with its typical half cylindrical morphology (see Figure 8a,d). In contrast to the equiaxed grains characterized for the top view (Figure 7), the majority of grains in the side view solidified in a columnar morphology, suggesting epitaxial growth, enabled by the lack of a nucleation barrier to solidification. These grains were elongated with their long vectors in parallel to (or slightly diverted from) the building direction. Others solidified with a fine equiaxed morphology and could easily be traced, as they were laced along the boundaries of the columnar grains. It is also worth mentioning the crystallographic texture distribution of the grains with respect to their relative sizes. The columnar grains were characterized by a cube texture component, while the fine grains were randomly oriented, as evidenced by various fine grain colors on the corresponding EBSD images (see Figure 8b,d). Therefore, this bi-model structure exhibited a bi-model crystallographic texture, which in turn contributed to the anisotropic properties of the SLM material, at least hypothetically.

As in the top view, a weaker cube textural component could be observed in the side view for the EOS sample, resulting from the scanning pattern. Figure 8c,f presents qualitative intra-grain strain analyses using the grain orientation spread (GOS) plots on the EBSD data recorded from the region of interest (ROI) indicated in Figure 8a,d. This type of map is color-coded using blue to red colors, which are correlated with the deformation severity for each grain. The assigned colors for an annealed grain, which lacks large deviation from its nominal crystallographic orientation; and for a deformed grain with a high density of the dislocation network, are blue and red, respectively. As can be observed in Figure 8c,f, both structures consisted of fine grains with a low GOS value, which point to their annealed state resulting from repeated local solidification. However, the elongated grains, with a columnar morphology, exhibited substantially higher GOS values and were therefore interpreted with a higher internal distortion. From the comparison of both plots, a further noticeable difference is the higher areal density of grains with a lower GOS in EOS compared with that of CL. That is, the EOS structure was significantly more stress relaxed. This can be associated with the higher solidification rate owing to the faster-moving laser source in the case of the CL.

### 3.2. Dynamic Mechanical Behavior

**Figure 9:**
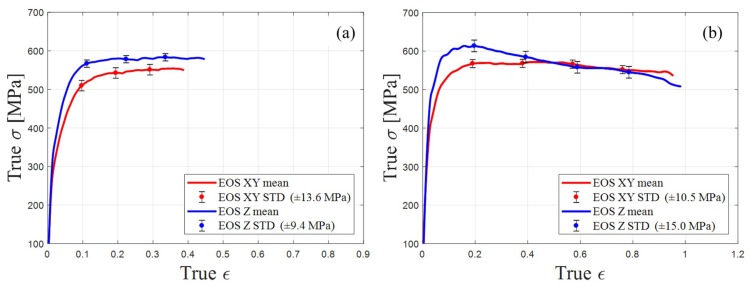
EOS samples mean value and standard deviation (STD) of true stress–true strain curves: (**a**) Comparison between XY- and Z-oriented samples at true strain rate ranged of 700 to 2800 s^−1^; (**b**) comparison between XY- and Z-oriented samples at true strain rate ranged of 4000 to 6700 s^−1^.

Figure 9 represents the mean values of the true stress–true strain curves obtained from the SHPB system. The results are from samples that were manufactured by the EOS machine. Figure 9a depicts the experiment conducted under strain rates ranging from 700 to 2800 s^−1^; while Figure 9b depicts the results under strain rates from 4000 to 6700 s^−1^. The strain is a function of the striker velocity, striker length, material, and geometrical properties of the bars and sample [33]. For the same set of geometric parameters, the experiment conducted with an increasing striker velocity resulted in an increasing strain rate, therefore increasing strain and stress in the samples. As reported in previous studies [8], the Z-oriented samples of EOS exhibit an approximately 10% higher yield stress level than the XY-oriented samples. This phenomenon refers to the point at which the sample begins its plastic failure (true ε ≈ 0.1). At true strain and higher, ε ≥ 0.4, it can be observed that the stress in XY-oriented samples remained constant and could endure the plastic deformation without reducing the stress. However, in the Z-oriented samples, higher stress levels were reached before plastic failure under the same conditions, but as the plastic deformation continued, the stress in the Z-oriented samples exhibited a decline, dropping below the stress level measured in the XY-oriented samples. In the studies of Asgari et al. [22] and Hadadzadeh et al. [23], the XY- and Z-oriented samples had the same dynamic behavior, and no significant differences were found in the stress–strain curves.

**Figure 10:**
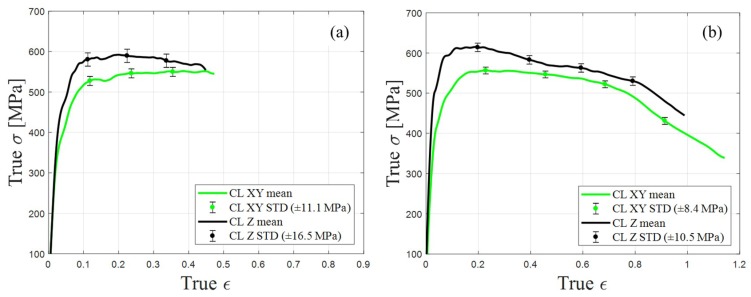
CL samples’ mean value and standard deviation (STD) of true stress–true strain curves: (**a**) comparison between XY- and Z-oriented samples at true strain rate range of 700 to 2800 s^−1^; (**b**) comparison between XY- and Z-oriented samples at true strain rate range of 4000 to 6700 s^−1^.

Similar SHPB experiments were performed with samples manufactured by CL, and the mean values of the true stress–true strain results are presented in Figure 10a,b for strain rates of 700 to 2800 s^−1^ and 4000 to 6700 s^−1^, respectively. Focusing on the plastic failure point (true ε ≈ 0.1), similar phenomena for the building orientation to those found in EOS were also identified in the samples manufactured by CL, and the stress value measured for the Z-oriented samples was approximately 10% higher than that of the XY-oriented samples. In the range of true strain ε = 0.35 to 0.8, the true stress characterization in the XY-oriented samples remained constant, while a decline was observed in the Z-oriented samples. As the true strain reached values of approximately 0.8 and higher (true ε ≥ 0.8), a sharp reduction in the true stress appeared in the XY- and Z-oriented samples manufactured by CL. This sharp decline is normally associated with cracks developing within the alloy (Section 3.3).

**Figure 11:**
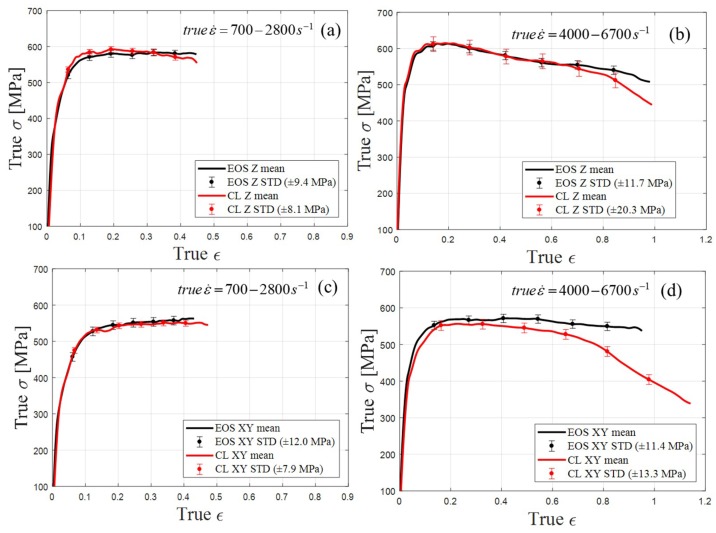
CL vs. EOS samples mean value and standard deviation (STD) of true stress–true strain curves: (**a**) Comparison between Z-oriented samples at true strain rate range of 700 to 2800 s^−1^; (**b**) comparison between Z-oriented samples at true strain rate range of 4000 to 6700 s^−1^; (**c**) comparison between Z-oriented samples at true strain rate range of 700 to 2800 s^−1^; and (**d**) comparison between XY-oriented samples at true strain rate range of 4000 to 6700 s^−1^.

To characterize the effect of the manufacturing on the dynamic behavior, this study implemented a comparison between the manufactures attributed to the building orientation and strain rate. Figure 11a,c depicts comparisons of the strain rates in the range of 700 to 2800 s^−1^ for the Z and XY orientations, respectively. In the presented strain rate range, a comparison between the two sample types (EOS and CL) revealed no significant difference in the strain–stress curves regarding the building orientation. This is valid up to a true strain of 0.5, as observed in Figure 11a for the Z orientation and in Figure 11c for the XY orientation (both under strain rates of 700 to 2800 s^−1^).

Figure 11b,d depicts the strain–stress curves in a strain rate range of 4000 to 6700 s−1. In Figure 11b, it is clearly observed that, up to a true strain of approximately 0.8, the two curves lay within each other’s standard deviation intervals. This suggests that a strong similarity existed between the EOS Z-oriented samples and CL Z-oriented samples up to a true strain of 0.8. The same applies to the EOS XY-oriented samples and CL XY-oriented samples up to a true strain of 0.6. It should be emphasized that higher internal distortion occurred with the CL, as presented in the GOS plots (see Figure 8c,f for CL and EOS, respectively). As GOS plots describe local characteristics on a micro-scale, the SHBP experiment—which reflected the bulk dynamic mechanical properties—lacked the sensitivity to emphasize those differences. Thus, minor differences that were rather insignificant were obtained in the maximum measured true stress and plastic behavior. In the XY orientation plot (Figure 11b) for a true strain higher than 0.8 up to the end of the measurement, a significant difference can be observed. A more significant difference appeared in the comparison of the XY orientation samples for a true strain of 0.6 and above (see Figure 11d).

The samples manufactured by the CL machine exhibited a decline in the stress–strain curve, which suggests that they experienced the same type of failure, while the samples manufactured using the EOS machine, where no declination occurred, experienced simple plastic deformation. The difference between the curves is beyond the standard deviation and measurement error. An analysis of the maximum true stress measured at the plastic failure point (*ε* ≈ 0.1) suggests that SLM–AlSi10Mg alloy has a very low strain rate sensitivity in contrast to previously reported results [22,24], and compared to precipitation-hardened aluminum alloys, where the increasing strain rate causes an increase in the yield point [34].

### 3.3. Post-Dynamic Loading Analysis

**Figure 12:**
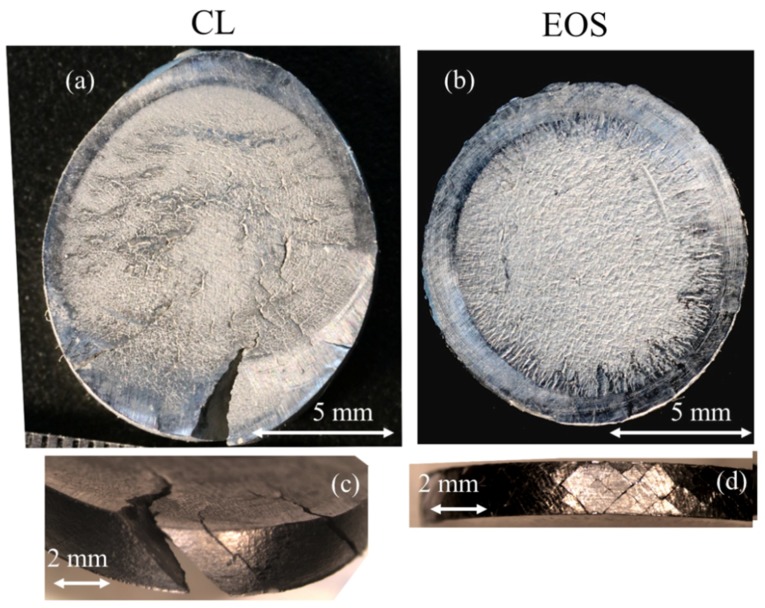
XY-oriented samples after SHPB tests at strain rate of 5800 to 6700 s^−1^, with plane view of samples for (**a**) CL and (**b**) EOS, and sample circumferences for (**c**) CL and (**d**) EOS.

**Figure 13:**
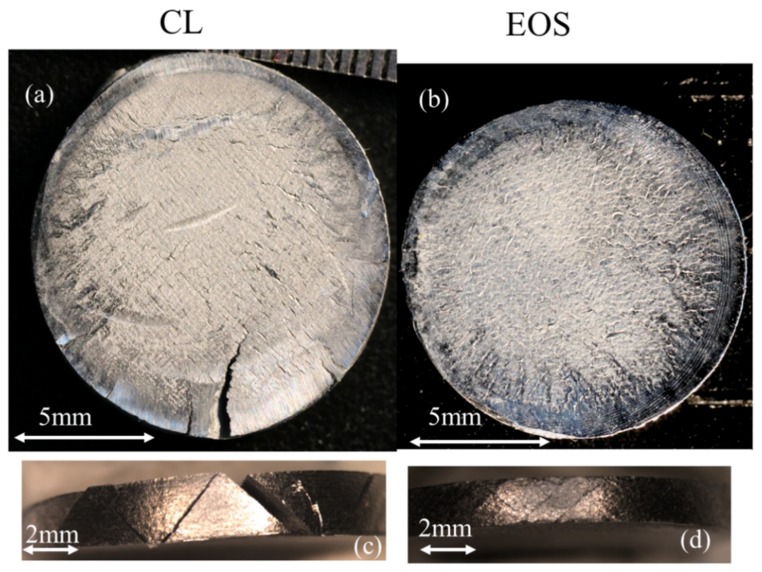
Z-oriented samples after SHPB tests at strain rate of 5800 to 6700 s^−1^, with plane view of samples for (**a**) CL and (**b**) EOS, and sample circumferences for (**c**) CL and (**d**) EOS.

The strain rate at a striker velocity of nearly 25 m/s was within the range of 5800 to 6700 s^−1^. For these samples, characterization of the damage was conducted using an optical microscope. Figure 12a,b depicts the EOS and CL XY-oriented samples viewed parallel and perpendicular to the loading direction. In the top view, it can be observed that both samples exhibited significant cracking, caused by severe plastic deformation. In Figure 12c,d, the circumferences of both samples are presented. These exhibited major brittle cracks inclined at 45° with respect to the loading direction. However, for the CL samples, the cracks along the circumference were notched throughout the sample and caused massive separation, as can be observed in Figure 12c. Moreover, the direction in which the cracks were formed was mostly one sided, namely at 45° with respect to the loading direction. In contrast, for the EOS sample in Figure 12d, the cracks along the circumference were inclined at 45° and −45°. Figure 13 illustrates the same phenomena of damage endurance between the EOS and CL samples in the Z-oriented samples. The EOS samples could endure plastic deformation by deflecting the cracks at the circumference in a twisted manner—see Figure 13b,d. The CL samples experienced massive failure when cracks propagated from the circumference into the center of the specimen—see Figure 13a,c. The unique crack nucleation and propagation could point to a different magnitude of anisotropicity inherited through each unique manufacturing strategy. As mentioned previously, the EOS product exhibited a weaker strength of the preferential textural component. It is plausible to assume that lower crack stability was expected for the EOS, as weak planes that pave crack paths are less likely to be continuous owing to the higher distribution of the crystallographic texture. Thus, when subjected to dynamic testing, the EOS will in turn endure damage more homogeneously, as indicated by the structure uniformity of the deformed samples (XY- and Z-oriented, Figure 12b,d and Figure 13b,d, respectively). However, for the CL, the dominant texture or, in other words, lack of local crystallographic alternation, resulted in a lower fracture resistance and higher crack growth, as can be observed in the CL deformed samples (XY- and Z-oriented, Figure 12a,c and Figure 13a,c, respectively), characterized by the spread of catastrophic macro-cracks.

## 4. Conclusions

SLM–AlSi10Mg alloy was tested for its dynamic behavior using the SHPB system, at strain rates ranging from 700 to 6700 s^−1^. The tested SLM samples were fabricated using the CL and EOS 3D printers, which are known for their unique manufacturing processes, mainly distinguished by their scanning pattern, scan speed, and beam energy. Hence, this fundamental study was established to relate dynamic properties to certain manufacturing processes.

The as-built state was characterized by its initial microstructure and crystallographic texture using the SEM–EBSD technique. While similar morphological features were observed in both SLM products, the magnitudes of their crystallographic texture and localized strain were somehow different. A preferential texture was more clearly observed for the CL product.

Under dynamic loading, the vertically built SLM–AlSi10Mg samples exhibited a higher yield stress compared to the horizontally built samples, regardless of the fabrication technique. During early compression strain, the samples individually manufactured by the two machines with the same building orientation exhibited the same stress behavior. However, at higher strains, a significant decline in stress was observed in the CL samples for both building orientations. The difference was much more pronounced in the XY orientation, and appeared at a smaller strain. This deference can be explained by the altered crystallographic texture caused by the manufacturing technique.

The compliance to deformation in the CL product when subjected to dynamic testing was obtained through crack nucleation and propagation, regardless of the building orientation. The damage endurance was traced to a higher magnitude of the preferential texture component for the CL product. The lower probability of local crystallographic alternation was plausibly manifested by continuous weak planes, which paved the crack growth path, and therefore resulted in a lower fracture resistance and higher crack growth in the CL product. However, the EOS product retained its structure without notable micro-crack formation, suggesting higher isotropic properties, as indicated by its lower preferential texture component.

## Figures and Tables

**Table 1:** Chemical composition, in wt.%, of AlSi10Mg alloy [27,28].

Al	Si	Mg	Fe	Mu	Ti	Cu	Zu
Balance	9–11	0.2–0.45	0–0.55	0–0.45	0–0.15	0–0.1	0–0.1

**Table 2:** Cumulative distribution of the AlSi10Mg alloy powder particle size and sphericity.

Manufacturer	Property	10%	50%	90%
EOS	particle size (m)	39.5	63.8	87.1
	sphericity	0.79	0.91	0.95
CL	particle size (m)	47.0	64.7	80.2
	sphericity	0.73	0.91	0.93

**Table 3:** Selective laser melting (SLM) process parameters as reported by manufacturers [27,28].

SLM Machine	Chamber Atmosphere	Build Platform Size (mm)	Focus Diameter (µm)	Laser Power (W)	Maximum Scanning Speed (m/s)	Layer Thickness (µm)
EOS M280	argon	250 × 250 × 300	80	400	1	~60
CL X line 1000R	nitrogen	630 × 400 × 500	100 to 500	1000	7	30 to 200

**Table 4:** Quasi-static tensile mechanical properties, as reported by manufacturers [27,28].

Manufacturer	Build Direction	Yield Stress (MPa)	UTS (MPa)	Elongation to Fracture (%)	Modulus of Elasticity (GPa)
EOS	Horizontal (XY)	270 ± 10	460 ± 20	9 ± 2	75 ± 10
	Vertical (Z)	240 ± 10	460 ± 20	6 ± 2	75 ± 10
CL	Horizontal (XY)	218 ± 7	345 ± 11	3 ± 1	75
	Vertical (Z)	214 ± 19	345 ± 8	3 ± 1	75
